# Working with national quality registries in older people care: A qualitative study of perceived impact on assistant nurses’ work situation

**DOI:** 10.1002/nop2.611

**Published:** 2020-08-31

**Authors:** Anna Westerlund, Vibeke Sparring, Henna Hasson, Lars Weinehall, Monica E. Nyström

**Affiliations:** ^1^ Department of Epidemiology and Global health Umeå University Umeå Sweden; ^2^ Department of Learning Informatics, Management and Ethics Medical Management Centre Karolinska Institutet Stockholm Sweden; ^3^ Centre for Epidemiology and Community Medicine (CES) Stockholm County Council Stockholm Sweden

**Keywords:** care of older people, national quality registries, quality improvement, team interaction, work environment

## Abstract

**Aim:**

The aim was to investigate assistant nurses’ perceptions of how working with national quality registries affected their work situation in care of older people.

**Design:**

Qualitative interview study.

**Methods:**

Sixteen semi‐structured interviews were conducted at four special housing units in Sweden, and a conventional content analysis, with elements of thematic analysis, was applied.

**Results:**

The introduction of national quality registries contributed to role clarifications and the development of new formal work procedures in terms of documentation and arenas and routines for communication. The increased systematics and effectiveness gained from these changes had a perceived positive effect on the work situation, workload, work satisfaction, staff interactions and learning and reflection.

## 1 INTRODUCTION

Quality assessment and improvement based on outcome measurements have increased in health care in general (Ellis, 2000; Evans, Scott, Johnson, Cameron, & McNeal, 2011; McNeil, Evans, Johnson, & Cameron, 2010). Such measurements make it possible for policymakers, managers, professionals, patients and their next of kin to monitor and compare care practices and health outcomes over time, and they can be used as the basis for continuous improvement (Ayers et al., 2005).

In the setting of Swedish health care, national quality registries (NQRs) have been developed to gather and structure data on patients’ problems/diagnoses, treatments/interventions and outcomes from care providers, nationwide (Emilsson, Lindahl, Koster, Lambe, & Ludvigsson, 2015). Beginning in the late 1970s, over 100 NQRs have been initiated and developed in an increasing pace by the professions. They have been used to a varied extent for three purposes: research, development of clinical practice and quality improvement. In the international literature, Swedish registry policies and NQRs are often cited as positive examples (Gray, 2013; James, 2003; Sousa, Bazeley, Johansson, & Wijk, 2006; Adami and Hernán, 2015,2015,2015). Sweden's structure for NQRs is well‐developed, and the Swedish model has been suggested as a precursor also for other countries (Levay, 2016).

Although the NQRs have had an increasing impact on quality and efficiency of health care in general (Levay, 2016), the phenomenon is relatively new in care of older people. A strong motive for seeking to improve quality and efficiency of care of older people is the increasing number of older citizens in the Western world, which will have doubled by the 2050 (United Nations, 2017).

In Sweden, we have seen an increased use of NQRs in care of older people since 2010 (Nyström, Strehlenert, Hansson, & Hasson, 2014). This study focuses on two NQRs especially suitable in care of older people, both with a preventive scope and a focus on risk assessments. The Senior Alert registry was initiated in 2008 and the Swedish Registry for Behavioural and Psychological Symptoms of Dementia (commonly referred to as the BPSD registry) in 2010. The Senior Alert registry gathers information on incidents and risks for falling accidents, malnutrition, decubitus ulcers and poor oral health and urine infections (Edvinsson, Rahm, Trinks, & Höglund, 2015). The BPSD registry registers information on psychological and behavioural disorders for people with dementia, for example hallucinations, sleep disruption, depression and support analyses and activity plans for patients. The Senior Alert registry has been investigated in recent years, mostly focusing on the assessments, the impact on analysis of risks and adverse events and on comparisons with health care (e.g. Johansson, Wijk, & Christensson, 2017; Trinks, Hägglin, Nordvall, Rothenberg, & Wijk, 2018). The dissemination of the registry (Nordin, Gäre, & Andersson, 2018a, 2018b) and the nursing staff's experiences of using a structured preventive care process advocated by the registry (Lannering, Ernsth Bravell, & Johansson, 2017) have also been studied. Due to the increased workload introduced by the registries, there are calls for more studies on the impact of Senior Alert (Lannering et al., 2017). There are far less studies on the use and impact of the BPSD registry (Bränsvik, Granvik, Minthon, Nordström, & Nägga, 2020).

## 2 BACKGROUND

Sweden has a high number of employees in care of older people in relation to the number of older citizens (Colombo, Llena‐Nozal, Mercier, & Tjadens, 2011). The work situation in care of older people is characterized by high psychological and physical demands, while competence levels among the staff members often are low (Hasson, 2006; Josefsson, 2012). Work‐related problems commonly reported by staff include heavy workload, high level of physical strain, time pressure and high turnover among managers and staff members (Hasson & Arnetz, 2008; Josefsson, Aling, & Östin, 2011; Westerberg, 2004). Job satisfaction and level of stress have been shown to have implications on the quality of care (Aiken, Clarke, Sloane, Sochalski, & Silber, 2002; Chou, Boldy, & Lee, 2003; Hannan, Norman, & Redfern, 2001). Furthermore, studies have indicated problems with creating continuous learning in care of older people (Hauer, 2013) and identified challenges in incorporating new innovative work methods or technical solutions (Andreasson & Winge, 2010). Thus, it has been challenging to achieve a combination of efficient and high‐quality services, a good work situation and an innovative friendly atmosphere in care of older people. Structural empowerment, with an opportunity to grow, adequate information, support and resources, has been suggested as an aid to improve the situation for managers and staff (Hagerman, Högberg, Skytt, Wadensten, & Engström, 2017).

It can be argued that the introduction of new work routines can affect staff's work situation in positive and negative ways. According to the job demand‐control model, an imbalance between perceived demands (e.g. workload, time pressure) and control (e.g. decision latitude, skills, competences) can increase stress, decrease work satisfaction and have a negative impact on employees’ health (Johnson & Hall, 1988; Karasek, 1979). Introducing NORs and related work procedures in care of older people may add new demands but may also provide a structure that increases control over the work situation and provide learning opportunities.

The consequences of working with the NQRs on the work situation are an important issue, especially in care of older people. Previous research on NQRs in health care has mainly focused on the effects of medical and care interventions in a disease area and/or on the care process and more recently on conditions for their use (Eldh et al., 2005; Granström, Hansson, Sparring, Brommels, & Nyström, 2018; Sparring, Granström, Andreen Sachs, Brommels, & Nyström, 2018). How staff's work situation is affected by work with NQRs has been less studied and no such study has focused specifically on care of older people. This study of the perceived impact of using a more structured preventive work process to improve care quality suggested by the Senior Alert and BPSD registries—on the assistant nurses’ work situation address this gap and combine the fields of quality improvement and work environment in care of older people.

Accordingly, the aim of this study was to increase knowledge of the relationship between work processes introduced by the NQRs in care of older people and staff's work situation. The main research question is What are assistant nurses’ perceptions of the impact of working with the preventive NQRs on their work situation in care of older people? The term “work with quality registries” refers to assessments, registration, analyses and planning for improvements, implementation of improvement efforts and follow‐ups on actions taken.

## 3 METHODS

### 3.1 Study design and participants

The study was grounded in the qualitative interpretative paradigm to enable addressing the study aims and providing a nuanced representation of the participants’ perspectives (Graneheim et al., 2017). The study draws on semi‐structured interviews with health professionals in four special housing units for older people. Convenience sampling was used. The selection of units was based on recommendations from a national network of regional improvement coaches in care of older people (all Swedish regions (*n* = 21) had at least one regional development coach employed during this period). A selection criterion was that the suggested units had been working with improvements based on NQRs for a minimum of 1 year. Based on the sample suggestions and on granting of access from higher level management, four special housing units geographically spread throughout the country were chosen. Two of the special housing units were large (six wards each), one was medium sized (with four wards) and one was small (two wards). Table 1 provides descriptive information about each municipality and the selected units.

**TABLE 1:** Overview of the municipalities and the selected special housing units

	Municipality A	Municipality B	Municipality C
Population	72,031	54,000	11,119
Special housing units	21	13	3 (+ 1 short‐term unit)
Number of residents	930	605	97 (+15)

	Unit I	Unit II	Unit III	Unit VI
Unit size	6 wards	2 wards	4 wards	6 wards
Number of unit managers	2	1	1	2
Number of informants (ass. nurses)	5	2	3	6
Specific patient characteristics	No	Severe dementia	No	Severe dementia

The study focuses on the largest professional group in care of older people in Sweden: assistant nurses and their perspectives on work with NQRs at their units. Assistant nurses work closest to the residents in special housing units. Nurses, rehabilitation staff and physicians often support several units and thereby meet individual residents less frequently. Participating assistant nurses were selected based on their length of involvement in work with the NQRs at the unit (ruling out staff who had not worked with the registries) and their availability during the interview period (convenience sample), ruling out a few assistant nurses, for example. on parental leave, leave of absence, holiday or studying. Between two and six assistant nurses from the four special housing units participated in the study (*N* = 16), with more informants from the larger units.

### 3.2 Data collection

Semi‐structured interviews were conducted in September and October 2015 with 16 assistant nurses. The interviews lasted between 30 and 60 min and focused on how work with the NQRs was organized at the unit; which factors were involved and in which role/function; and perceived effects of work with the registries on workload, work satisfaction, learning and reflection (henceforth, we use the concept “work situation” when collectively referring to these aspects). All interviews were audio recorded and transcribed verbatim.

### 3.3 Data analysis

**FIGURE 1:**
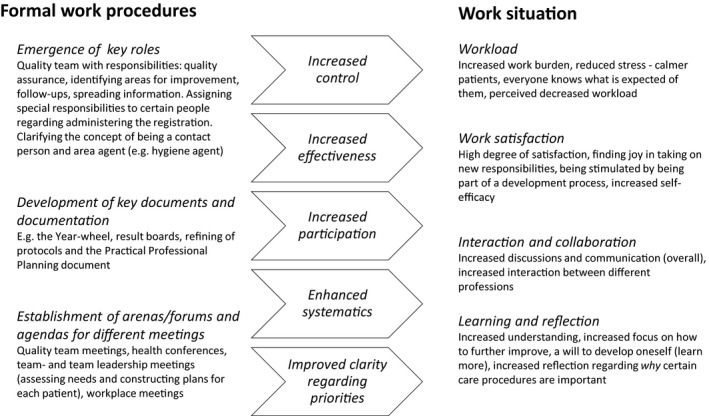
Overview of the main impact of working with quality registries on formal work procedures and how this affects the assistant nurses’ work situation

A conventional content analysis, with elements of thematic analysis, was conducted in four steps (Hsieh & Shannon, 2005). The procedure started with a reading of all transcripts to obtain a sense of the whole. In the second step, descriptions of how work with the NQRs was organized at the unit (i.e. how the registries had affected formal work procedures) were identified, condensed, coded and then categorized. In a third step, meaning‐bearing segments of text about perceived effects of work with the two registries on areas related to staff's work situation (ranging from a single phrase to several paragraphs) were condensed to a few words/codes and sorted into categories. Thereafter, all the material was coded in the same manner, focusing on relationships between new work procedures as a result of the introduction of the NQRs and the effects on the assistant nurses’ work situation that were identified. This resulted in themes describing the type of relationships between two overarching categories of perceived effects of work with NQRs: (1) formal work procedures and (2) work situation, with sub‐categories covering all relevant aspects of the manifest content. An overview of the organization of codes, categories and themes is given in Figure 1. The analysis was performed by the first author and regularly checked by and discussed with the last author. Saturation was assessed by three researchers (first, second and last authors) and compared with information in documents and in interviews with nurses, rehabilitation staff and unit managers conducted at the same time for another study. At two occasions, one more interview was performed when staff returned to their work.

### 3.4 Ethics

All participation was based on informed consent. Information about the study, contact persons and how confidentiality would be ensured was provided, first via e‐mail to the unit manager (to inform to everyone involved in work with the NQRs at the unit) and then verbally and in a document given to each participant before the interview started. Ethics approval was obtained from the Regional Ethics Committee in Stockholm (no. 2011/1598‐31/5).

## 4 RESULTS

The results on the assistant nurses’ perceptions on work with the NQRs are presented in accordance with the categories and themes identified, and the relationship between the two main categories, formal work procedures and work situation, is identified. Some differences in the findings for the four special housing units are also presented. Figure 1 provides a summary of the main results about the perceived impact of work with NQRs on formal work procedures and on the assistant nurses’ work situation.

### 4.1 Impact of work with quality registries on formal work procedures

Work with the NQRs was found to have an impact on formal work procedures by initiating establishment and/or development of (1) key roles for both individuals and groups; (2) documentation; and (3) communication arenas and agendas for meetings.

#### 4.1.1 Establishment of key roles

At Units I and II (municipality A), a central change was the formation of a quality team, which includes members from different categories of staff (unit manager, nurse, physiotherapist and assistant nurses). The quality team has been given responsibility for ensuring the quality of care at the unit and the task of continuously identifying new areas of improvement. They have also been assigned responsibility for disseminating information and new ideas to the rest of the staff, and analysing results from registries and gathering information that could inform decisions on improvement measures needed.

At all units, the role and responsibilities of a contact person have been clarified. One of the main responsibilities of a contact person is to identify risks and areas in need of improvement for each resident (e.g. conducting risk assessments on an individual basis before registration). At Units I and II, specific BPSD administrators (assistant nurses) have been trained to be responsible for registrations in the BPSD registry. For Senior Alert, each contact person is responsible for performing risk assessments and the registrations are conducted by the nurse.

At Unit III, all assistant nurses share the responsibility for identifying risks and needs for improvement for all residents. This is achieved in dialogue among the staff, but the contact person is ultimately responsible for the documentation and implementation of actions concerning each resident. The contact persons collaborate on a regular basis with the nurse in the unit about assessments, registrations and the implementation of improvement measures. At Unit III, some assistant nurses with special responsibility for monitoring new knowledge in certain assigned areas (e.g. hygiene) had a central role to provide information and knowledge in their areas. This information was considered when deciding on and designing improvement actions.

At Unit IV, the major reasons behind successful work with the NQRs highlighted by all respondents were the development and clarification of the roles of the team (all staff represented) and of the management team (i.e. unit manager, the unit nurse and occupational therapist).

#### 4.1.2 Development of key documents and documentation

The development of existing documents and documentation routines was another effect of working with the national NQRs that was common to all units. Unit I created a result board displaying aims and results over time related to different areas measured in registries; the board presented an overview of improvements over time.

At Unit II, respondents highlighted the development of the documentation structure, practical professional planning, as a major improvement. Here, everything concerning a resident is documented and all staff are responsible for regularly keeping themselves informed of what is written in the documentation structure. Respondents stated that this was a key document and a way of communicating and improving.

At Unit III, the risk‐assessment protocol (underlying registrations) was a key document and the basis for discussions at different meetings. Another important way of sharing information mentioned was the systematic documentation of health status and improvement measures for each resident in the medical record Pro‐capita.

At Unit IV, the development and use of the so‐called year wheel was a central effect of the initiation of work with the NQRs. The year wheel clarifies when registrations (and risk assessments underlying these registrations) and follow‐ups should be conducted for each resident. Protocols from team meetings were also mentioned as an important and useful document.

Common to all units and mentioned by all respondents were the use of (slightly different) implementation plans, that is documents that clarified overall goals and measures related to each resident. This document also guided the follow‐ups. Respondents at Units II and III stated that these implementation plans had become much more useful and person centred because of the work with the registries.

#### 4.1.3 Establishment of communication arenas and agendas for different meetings

A commonly mentioned consequence of working with the new registries was increased clarity about when and why different meetings were scheduled and who should participate. At Units I and II, the most central change was the occurrence of quality team meetings, which at the time of the study took place twice a month. The main purpose of quality team meetings was to analyse results from registries, discuss how to ensure quality and plan for improvements.

At Unit III, the monthly health conference was a central forum for analysing results, planning for improvements and following up on certain measures. Respondents stated that the whole design of nursing and care was developed at the health conference. At Unit III, the care, nursing and improvement measures were highly individualized, that is discussions were held for each resident based on the NQRs’ risk‐assessment protocols.

At Unit IV, matters related to work with NQRs were discussed at team meetings and team leadership meetings. All staff were represented (assistant nurses, nurse, unit manager, physiotherapist and occupational therapist) at the weekly team meetings where the discussion and analysis of results, and follow‐ups on health effects and identification of new improvement areas took place. At the monthly team leadership meetings (unit manager, nurse, occupational therapist), specific improvement measures were decided on and planned for and then incorporated into the implementation plans. All respondents from all units also mentioned the regular workplace meetings as a central arena for information sharing and discussions related to work with the NQRs.

### 4.2 Effects on work situation

#### 4.2.1 Perceived workload

Respondents from all units generally described an increased workload because of work with the registries. Performing assessments and registrations in the registries added a time‐consuming task to their regular work routines, a task that also required certain computer skills. However, the clarification of work roles together with enhanced systematics stemming from work with NQRs and clarity about jointly discussed decisions on improvement measures led to a perception of a decreased workload and reduced stress, despite the addition of new assessment and registration tasks:

The things we need to do and not do is clear now. It becomes clear when we analyse the results (of registrations). No one needs to run around and wonder what to prioritize – what should I do and not do…? (Unit IV).

At Units I and II, the increased workload for members of the quality team was mentioned and the unit managers’ expectations on what the quality team should accomplish were mentioned as being a bit unrealistic:

I believe our managers expect more of us than we can deliver, a little bit too high expectations on what you can accomplish. You cannot show up and state to a group – now we are going to do this and that, etc. Such an approach could easily backfire. (Unit I).

At Unit II, respondents expressed a feeling of reduced stress mainly as a result of the development of documentation structures:

The development of the documentation structure, Practical Professional Planning, has rendered a major change! Everyone feels a lot less stressed now, because the clarifications make us feel that everyone can individually plan their own day based on their assignments for the day. (Unit II).

I experience a calmer situation [at the unit]. Even though it is a lot of work, you still experience a kind of calmness. Yes, we perform the registrations and so on, but we have found a harmony in that; it comes naturally. (Unit II).

Respondents from Unit III perceived that as a result of the improved health conferences, priorities on what should be discussed had been clarified and the possibility of affecting and controlling the work situation had increased:

Well, it becomes better because you have a feeling of being in control. I can affect my work situation. (Unit III).

At Unit III, one respondent highlighted the dilemma of how work with the registries competed with the daily caregiving procedures:

Sometimes one can feel that it is a lot [of work] to have to write a lot, that this takes time from actual care. (Unit III).

All respondents from Unit IV described the clarification of roles and responsibilities and the documentation in the year wheel as stress‐reducing factors:

Every month, I know what to do with the different residents that I, as a contact person, am responsible for. I know when it is time for Senior Alert or BPSD. I never have to think about it or wonder. I get it in black and white and no one is forgotten. (Unit IV).

Respondents from Unit IV also mentioned that work with the registries initially had a negative impact on workload, but over time—due to increased learning and increased clarity—the impact on workload was perceived as positive.

#### 4.2.2 Work satisfaction

Respondents at all units described mainly positive effects of work with the NQRs on work satisfaction. Respondents valued the core elements of working with the registries, such as risk assessments, registrations, analyses of results and follow‐ups, as relevant and necessary and as a facilitating factor in relation to the aim of providing high quality of care. The work with the NQRs and the increased sense of control it provided also seemed to have contributed to better self‐esteem when dealing with care situations:

We know what we are doing and why. We get it in black and white now and nothing gets overlooked; and this is highly motivating. To know when I go home that I have done a good job! (Unit IV).

I really enjoy the way things have changed. I think it is stimulating to find new ways and when you measure it and follow‐up on it systematically, you can see that you have done the right thing and that it is good for the patient. (Unit II).

At Unit I, members of the quality team stated that involvement in the development process was stimulating and receiving new responsibilities was perceived as an opportunity and as a rewarding possibility to affect their own work situation. Respondents from Unit II pointed out that it was highly motivating to see the good results of the work with the NQRs, but also mentioned the problem of people resisting change and development.

At Unit III, respondents found that work with the registries had contributed to the daily work routines and the development and refinement of current work procedures and that this was stimulating:

The positive thing is that I know exactly what should be done now and I know what to expect from the others and everyone knows what they are supposed to do. But the negative part is the stress that arises when you notice that some people just do not do things the way you have agreed on. (Unit II).

#### 4.2.3 Learning and reflection

Most of the respondents expressed increased learning and reflection as a result of working with the NQRs and increased willingness to learn new things by taking on more responsibilities. An extended holistic understanding of the care situation at the units was highlighted, and increased reflection due to the clear and available documentation:

The holistic understanding has increased, as well as the motivation to take on more responsibilities, which is great! (Unit IV).

The result board on the wall creates lots of reflection. If you study it, you can see results from each department and then you might discover that another department has a better score on, for example, nutrition and that makes you wonder – what can we do to get better at this? (Unit I).

At Unit II, respondents mentioned the increased learning resulting from more collaboration between the different professions:

If we say: “Shall we do some training by walking with someone?” Then the nurse comes and helps us by showing how and we make up a program. Often, a few staff learn how and then we can learn from each other. (Unit II).

Respondents from Unit II highlighted the increased knowledge and reflection related to the areas measured by the registries. At Unit IV, some respondents discussed the increased reflections about the content covered by Senior Alert and BPSD, for example nutrition, falling accidents, and oral health, whereas some respondents did not perceive any effects on learning and reflection at all:

Previously you may not have thought of malnutrition or falling incidents for over a year. Now you think about it all the time, even if you do not have any resident with high risks in those areas. (Unit II).

There is an increased reflection concerning why certain care procedures are important. (Unit IV).

#### 4.2.4 Collaboration and interaction

Results from all units indicate that working with the NQRs and the focus on teamwork that followed generated increased interaction among and between different professions and rendered a more open climate at the units:

Before, you had to run around asking people if they need help. Now people just ask each other more openly. No one needs to wonder if anyone needs help because in that case – they will ask for it. This has reduced the running around a lot! (Unit II).

Respondents from all units also pointed out that clarification of the contact person's role led to increased understanding of other staff's situation, and an overall level of involvement and collaboration:

The changes in the contact person role that are directly and indirectly connected to the registries has led to increased understanding of each other (staff) and increased involvement and collaboration. (Unit III).

At Unit I, collaboration and communication with staff working nightshifts had improved, mainly as an effect of the focus on nutrition (i.e. the increased dialogue and follow‐ups between night and day activities concerning nutrition).

Respondents at Unit I involved in the quality team pointed out some difficulties about communication and the spread of information from the quality team to the rest of the staff:

Sometimes it's hard to spread our [quality teams’] ideas, because – as with any change effort – it is hard for some people. They do not really want to listen and the comprehensive view of the situation we gain in the quality team… I am not sure everyone gets that. (Unit I).

At Unit II, respondents pointed out that collaboration had increased a lot, also across professions and that work with the NQRs was dependent on such collaboration. Team meetings were described as a key arena for this:

We are collaborating more regarding everything, among professions and over the entire care chain. So, it is getting better, also among staff – because we must collaborate to make this work. (Unit II).

Respondents from Unit III highlighted that the increased structure for communication about risk areas and dealing with various care situations and residents had led to increased and more efficient collaboration:

I feel that the collaboration with the nurse and the rehabilitation staff has improved and that we have the same goals. We all meet during the health conferences, which I think is good and this has improved the contact also between the meetings. (Unit III).

At Unit IV, awareness of the importance of teamwork increased and teamwork developed, as well as team meetings. Respondents also expressed increased understanding of each other and increased dialogue with staff working nightshifts:

You feel that we are a team together with the nurse and rehabilitation staff, because we work together. It is not us and them. (Unit IV).

## 5 DISCUSSION

Introducing the NQRs led to three important changes about formal work procedures at the units: the emergence of key roles, the development of key documents and documentation procedures and the establishment of key arenas, forums and agendas for different meetings. The enhanced systematics, increased effectiveness and clarification of role expectations and priorities gained from these changes in work procedures and routines had in turn a positive effect on staff's perceived workload, work satisfaction, collaboration, interaction, learning and reflection.

The main results are discussed in relation to the job demand‐control‐support model (Johnson & Hall, 1988). The hypothesis underlying the job demand‐control‐support model implies that employees working in a high‐strain job (i.e. high demands alongside low control and low social support) experience the lowest well‐being. Control and social support are hypothesized to be able to moderate the negative impact of high strain on well‐being (Van der Doef & Maes, 1999). Optimal use of NQRs for the improvement of care and patients’ well‐being places high demands on competence, knowledge and understanding and adequate formal work procedures. An interesting paradox in the findings is that although respondents expressed that their actual workload (demands) had increased, they reasoned that the positive effects of work with NQRs resulted in a perceived decrease in workload.

Working with the NQRs was perceived to have led to increased control, both in terms of increased learning and reflection (knowledge) and by aiding structure and a systematic way of identifying, acting on and monitoring quality deficiencies. Work with the NQRs was more a shared activity than a single individual's responsibility in these units, even though there were some specific responsibilities tied to different functions and roles. Work with registries seems to have been related to increased collaborative efforts and to have benefitted staff interaction and teamwork. The increased teamwork across professions enhanced learning and facilitated a continuous process of building competence. Thus, the paradox of the actual increased workload (i.e. increased demands), being perceived as decreased, may be explained by the strain hypothesis presented in the job demand‐control‐support model. The perceived increased control (knowledge and systematics) alongside the increase in interaction and collaborative efforts (social support) moderated the impact on strain related to work with the NQRs. In addition, quality registry work helped clarify priorities.

Another positive impact of working with the NQRs seems to have been a clarification of role expectations. It is important for a good work situation to avoid both quantitative and qualitative role overload (Savelsbergh, Gevers, van der Heijden, & Poell, 2012) and clarification of priorities and responsibilities together with increased knowledge might have reduced this risk at the units. The development of a clear role distribution is also likely to have enhanced teamwork and collaboration, which increased at the units, an assumption also supported by other researchers (Blickensderfer, Cannon‐Bowers, & Salas, 1997).

### 5.1 Strengths and limitations

To increase the credibility and confirmability of the study, data collection was performed by several researchers and coding strategies and interpretation of the data and the emerging findings were discussed in the research group. Quotes were presented in relation to the results and the similarity of issues raised by the informants also strengthens the trustworthiness of the findings. The fact that the research group had multidisciplinary backgrounds also strengthened the possibility to understand data from different perspectives. To increase dependability, data analysis adopted a coherent and systematic procedure.

The study is limited to a sample of units that were judged by regional development coaches (accessed via their national network governed by the Swedish Association of Local Authorities and Regions) to have well‐developed approaches on working with the NQRs. This sampling procedure deviates from a previous study with random sampling of nursing homes, presenting fewer positive results on experiences of working with Senior Alert in 2015 (Lannering et al., 2017). Results from the present study are positive overall and somewhat homogeneous. It is important to investigate further and outline the challenges and impact related to working with NQRs at multiple units with diverse local conditions and at different stages of establishing related work procedures. Using interviews was appropriate in this exploratory study. Mixed methods with validated questionnaires measuring workload, etc. can be used to further investigate relationships between formal work procedures, procedures for working with NQRs (or similar) and aspects of the staff's work situation in care of older people and thus include a larger sample of units and staff.

### 5.2 Conclusions

The NQRs were mainly developed to increase the quality of care. In addition to the intended main effects (i.e. enhancing the development of high‐quality care of our oldest citizens), work procedures introduced when working with the two preventive registries also have the potential to contribute to a positive work situation for assistant nurses in care of older people, by clarifying roles, increasing collaboration, interaction, learning, reflection and their sense of control. This in turn may have positive effect on their perceived workload and work satisfaction. The study is unique as the NQR’s potential to affect staffs’ work situation in health and social services has not been studied previously. Further research on how different types of quality registries and the work procedures they introduce affects employees’ work situation and the improvement of care in different areas is recommended.

## CONFLICT OF INTEREST

The authors declare that they have no competing interests.

## Data Availability

The qualitative data for this study are safely stored by the Department of Epidemiology and Global health, Umeå University. It consists of transcripts from semi‐structured interviews (all text in Swedish). It is available on request to the first author, after signing appropriate documents in line with the ethical application and Ethics Board's decision. Excerpts from the data sources are presented in the article as citations (translated to English).
